# Association between folate intake and risk of head and neck squamous cell carcinoma: An overall and dose–response PRISMA meta-analysis

**DOI:** 10.1097/MD.0000000000008182

**Published:** 2017-10-20

**Authors:** Chengchao Fan, Siwei Yu, Si Zhang, Xiaojun Ding, Jian Su, Zhigang Cheng

**Affiliations:** Department of Oral and Maxillofacial Surgery, The Central Hospital of Wuhan, Tongji Medical College, Huazhong University of Science and Technology, Wuhan, China.

**Keywords:** dose–response, folate, HNSCC, meta-analysis

## Abstract

The results of published studies about the relationships between folate intake and risk of head and neck squamous cell carcinoma (HNSCC) remained inconsistent. Hence, a comprehensive and dose–response meta-analysis was performed to clarify the association between folate intake and HNSCC risk.

The electric searches of Pubmed, Medline, and EMBASE databases were performed to identify the studies examining the relationship between folate intake and HNSCC risk on April 5, 2017. According to the inclusion criteria, finally 9 studies were included in this meta-analysis. The pooled odds ratios (ORs) with 95% confidence intervals (CIs) were used to evaluate the strength of associations. Dose–response analysis was conducted to quantitate the relationship between dietary folate intake and HNSCC risk.

The pooled OR for assessing the risk of HNSCC and folate intake in the highest level versus lowest level was 0.505 (95% CI 0.387–0.623). The linearity model of dose–response analysis indicated that with increased 100 μg/d folate intake, the risk of HNSCC decreased 4.3% degree (OR 0.957, 95% CI 0.935–0.980).

These results indicate that folate is a protective nutrient against HNSCC carcinogenesis.

## 1 Introduction

Head and neck squamous cell carcinoma (HNSCC), which is the sixth incidence of malignance worldwide, causes about 600,000 new cases and leads about 200,000 deaths annually.^[1,2]^ Although treatments for HNSCC have been developed rapidly recently, the 5-year survival rate of patients is only 40% to 50%.^[3]^ Therefore, efforts toward modifiable risk factors and primary prevention have become paramount importance.

The fact that low levels of vegetable and fruit intake are associated with increased risks of HNSCC suggests that folate, which is abundant in vegetables and fruits, is a protective nutrient for HNSCC.^[4,5]^ Folate, also called vitamin B9, plays an important role in the process of DNA synthesis, repair, and methylation.^[6]^ Low level of folate attenuates DNA methylation and promotes carcinogenesis.^[7]^ By decreasing thymidylate biosynthesis de novo, folate deficiency causes mis-incorporation of uracil during DNA repair and synthesis, and induces DNA strand breaks, and ultimately results in malignant transformation.^[8]^

Many studies have summarized previous published data and indicated that increased folate intake was associated with the increased risks of prostate^[9]^ and breast^[10]^ cancers, but decreased the risks of colorectal,^[11]^ oropharyngeal,^[12]^ esophageal,^[13–15]^ pancreatic,^[13,14,16]^ and cervical^[17]^ cancers. Almadori et al^[5]^ firstly reported a positive relationship between serum folate level and HNSCC risk. Then other epidemiologic studies have estimated the associations between folate intake and HNSCC risk.^[18–21]^ However, the role of folate intake level in HNSCC has remained controversial. Hence, a comprehensive meta-analysis was conducted to determine the link between folate intake and HNSCC risk, and to evaluate the dose–response relationship between folate intake level and HNSCC risk.

## 2 Materials and methods

This meta-analysis was performed according to the latest Preferred Reporting Items for Systematic Reviews and Meta-Analyses (PRISMA).

### 2.1 Literature search

A systematically search was performed up to April 5, 2017 by 2 reviewers (CF and SZ) within Pubmed, Medline, and EMBASE, using the following search strategy: ((((folate OR folic acid OR vitamin B9))) AND ((head and neck squamous cell carcinoma OR HNSCC OR oral OR laryn∗ OR pharyn∗ OR tongue OR oropharyn∗ OR nasopharyn∗ OR hypopharyn∗ OR trachea OR laryngopharyn∗ OR cervical tracheal OR cervical esophagus))) AND ((cancer∗ OR tumor∗ OR carcinoma∗ OR neoplasm∗)). In addition, the reference lists from original reports were reviewed and manually selected for other available publications. No language restrictions were imposed in the searching process.

### 2.2 Study selection

The inclusion criteria of the included studies were as followed: the experimental design was a case-control or cohort study; studies reported the associations of histological diagnosed HNSCC risk with dietary folate intake from diet and serum levels of folate; relative risk (RR), hazard ratio, or odds ratio (OR) with 95% confidence interval (CI) was given to estimate the association of the highest folate intake versus lowest folate intake; for dose–response analysis, the number of cases and participants, and eligible dose concentration must be provided. The selected studies were only limited in using dietary folate intake as only measurement standard.

The most recent study was included for duplicate publications.

### 2.3 Data extraction and quality assessment

Data were extracted independently by 2 authors (SY and CF). The following information was selected according to the criteria listed previously: the first author's name, publication year, country, study design, total sample size (cases and controls), difference between highest and lowest folate levels, measurement, range of exposure, adjusted variables, risk estimates, and 95% CI for evaluating the highest folate levels versus lowest folate levels. The adjusted ratios that had been maximally adjusted for potential confounders were chosen when studies reported several multivariable adjusted effect estimates. All controversial questions were resolved by asking a third author.

We assessed study quality using the Newcastle–Ottawa quality assessment scale (NOS) system, which has been validated as a comprehensive tool for assessing the quality of observational studies in meta-analysis.^[22,23]^ NOS evaluating details including the following 3 subscales awarded a maximum of 9 items: selection of participants and measurement of exposure (4 items), comparability (2 items), and evaluation of methodological quality outcome (3 items). Studies with 7 score or higher score were considered as high-quality studies.^[15,24]^

### 2.4 Statistical analysis

Pooled risk estimates (RR or OR) with 95% CI were used for detecting the associations of folate intake with risk of HNSCC. The heterogeneity test was evaluated with *I*^2^ statistic. Cut-off points of *I*^2^ value were for 25%, 50%, and 75% for low, moderate, and high degrees of heterogeneity, respectively. When heterogeneity was negligible, a fixed-effect model was chosen; otherwise, the random-effect model was chosen.^[25]^ Sensitivity analysis was performed to evaluate robust of pooled results by omitting 1 study each time when heterogeneity was significant. The publication bias was determined with the Begg rank model and Egger linear model.^[26]^ A stratified analysis was performed by study location, anatomical site, source of control, folate assessment, and study quality. At last, a dose–response meta-analysis was conducted by using the correlated natural logs of the RRs or ORs with their standard error (SE) across all folate intake categories. To drive the dose–response curve, we used the restricted cubic splines with 4 knots at the 5%, 35%, 65%, and 95% percentiles of the distribution to evaluate the potential curvilinear relations.^[27]^ All statistical analyses were performed using Stata 12.0 (StataCorp LP, College Station, TX).

## 3 Results

### 3.1 Summary of study characteristics

**Figure 1:**
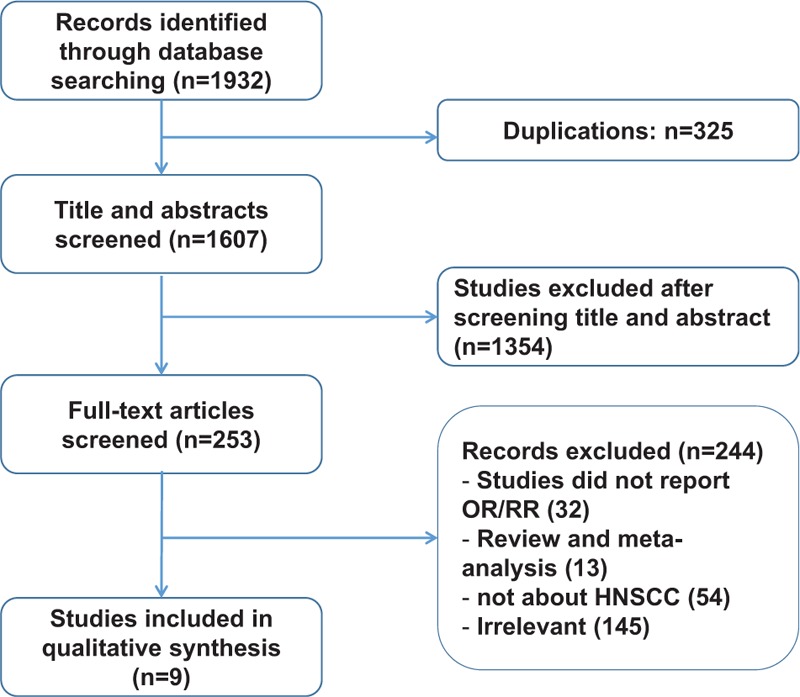
The flow diagram of the literature search, analysis and excluding used in this meta-analysis.

After duplicates removing within EndNote, total 1607 articles were identified from our initial search. Then, 1354 studies were excluded after screening titles and abstracts; the remaining 253 studies were evaluated eligibly with full text reading. Lastly, according to our inclusive criteria mentioned in “Materials and methods” section, 9 studies were included in our meta-analysis. The search results and eligible literature selection process are shown in Fig. 1.

The included main studies were conducted in Europe (n = 5),^[18,21,28–30]^ and others were conducted in Asia (n = 2),^[20,31]^ North America (n = 1),^[19]^ and South America (n = 1).^[32]^ Among the 9 included studies, 3 studies involved patients with comprehensive HNSCC,^[19–21]^ 3 studies involved patients with laryngeal cancer,^[18,29,32]^ 2 studies involved patients with nasopharyngeal carcinoma,^[30,31]^ and 1 study involved patients with oral cavity and pharyngeal squamous cell carcinoma (OPSCC).^[28]^ Seven studies investigated the associations between dietary folate intake and HNSCC risk.^[18,20,28–32]^ Two studies evaluated the links between serum folate concentration and HNSCC risk.^[19,21]^ In terms of source of control, 7 studies were hospital-based^[18,28–32]^ and 2 studies were population-based.^[19,21]^ Five studies matched high score with NOS scale,^[18–21,31]^ and 4 studies matched low score.^[28–30,32]^ Four studies were included in dose–response analysis.^[20,29,31,32]^ The main profiles of the included 9 studies are summarized in Table 1.

**Table 1:**
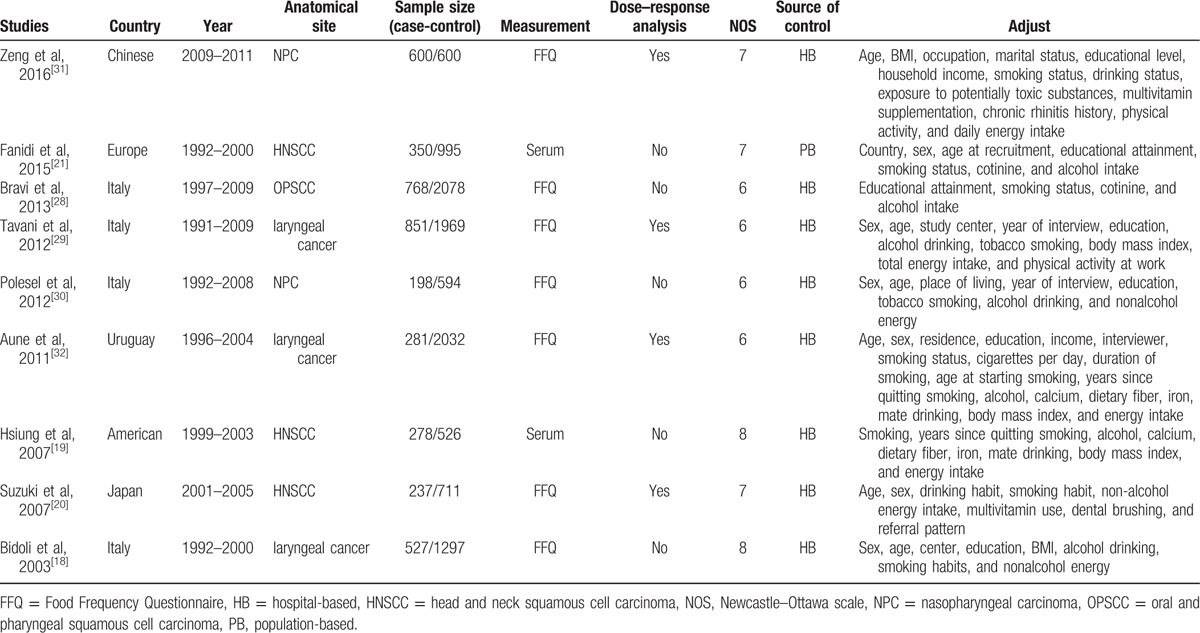
Characteristics of studies included in the meta-analysis.

### 3.2 Dietary folate intake

**Figure 2:**
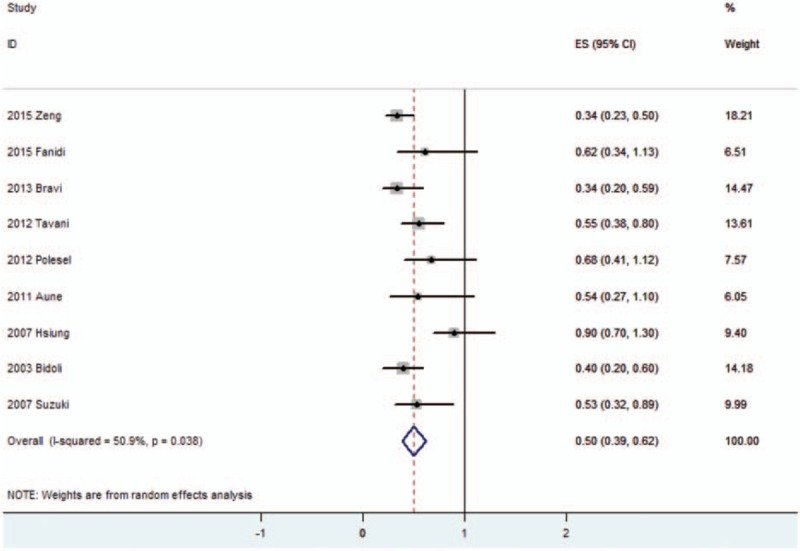
Forest plots of the association between dietary folate intake and risk of head and neck squamous cell carcinoma.

To evaluate the link between dietary folate intake and HNSCC risk, total 9 case-control studies including 4090 patients and 10,902 controls were collected. The significant heterogeneity (*P* = .038, *I*^2^ = 50.9%) suggested that a random-effect model was available. The pooled ORs of HNSCC for highest levels versus lowest levels was 0.505 (95% CI 0.387–0.623; Table 2, Fig. 2), which suggested a significant association between increased dietary folate intake and decreased HNSCC risk.

**Table 2:**
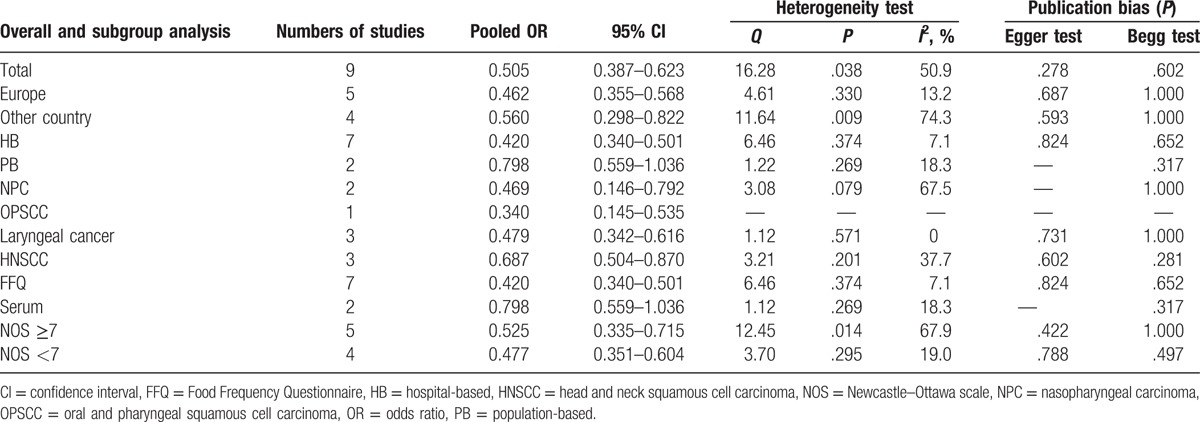
Results including overall and subgroup analysis of pooled OR, 95% CI, heterogeneity test and publication bias.

Table 2 shows the detailed results of specific subgroup analysis stratified based on countries, anatomical sites, source of controls, folate intake measurement, and NOS quality. All these results were similar and suggested inverse links between dietary folate intake and HNSCC risks for all the analyzed subgroup strata.

### 3.3 Dose–response analysis

**Figure 3:**
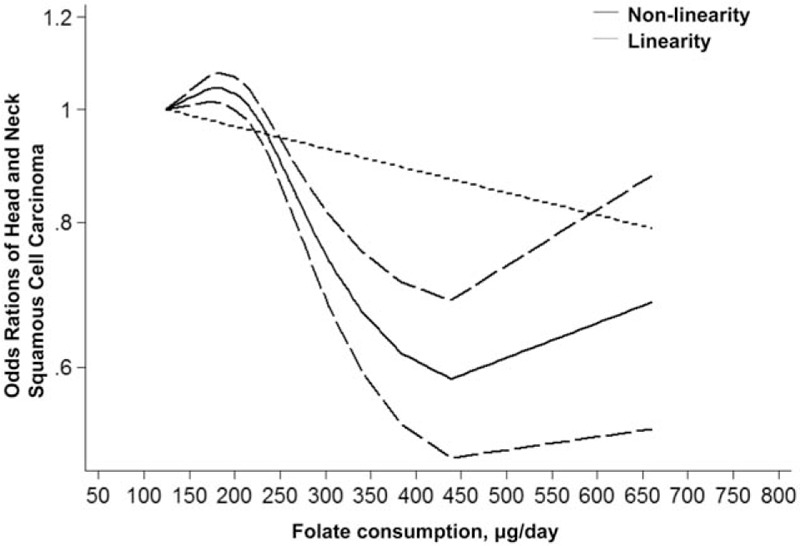
Linearity and nonlinearity relationships between dietary folate intake and risk of head and neck squamous cell carcinoma.

To determine the relationship between dietary folate intake and HNSCC risk, a dose–response analysis including 4 case-control studies was performed. As shown in Fig. 3, the linearity test of dose–response analysis suggested that with increased 100 μg/d folate intake, the risk of HNSCC decreased 4.3% degree (OR 0.957, 95% CI 0.935–0.980). The nonlinearity test also indicated an inverse association between dietary folate intake and HNSCC risk (*P* < .001).

### 3.4 Sensitivity analysis and publication bias

**Figure 4:**
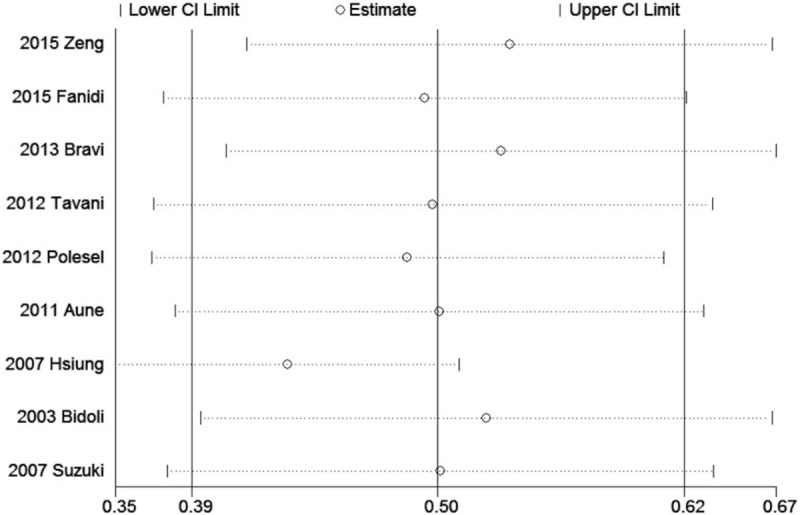
Sensitivity analysis of the pooled odds ratios (ORs).

High heterogeneity suggested a sensitivity analysis to be necessary. As shown in Fig. 4, the sensitivity analysis was performed by omitting 1 included study each time and showed stable results in this meta-analysis. The publication bias was evaluated by Begg test and Egger test, and the results were shown in Table 2. No significant publication bias was detected in this meta-analysis.

## 4 Discussion

Folate plays an important role in the process of DNA synthesis, repair, and methylation.^[33,34]^ Folate intake was assumed to be protectable against varieties of cancers including colorectal,^[11]^ oropharyngeal,^[12]^ esophageal,^[13–15]^ pancreatic,^[13,14,16]^ and cervical^[17]^ cancers. Eighty-two countries have passed legislations that mandate folate fortification in at least 1 industrially milled cereal grain on December 2014.^[35]^ However, conflicting studies have summarized published results that indicated a positive relationship between folate intake, and prostate^[9]^ and breast^[10]^ cancers, which suggests different role of folate in cancer prevention. To our knowledge, no study has investigated the relationship between folate intake and HNSCC risk. Hence, we conducted a comprehensive meta-analysis to evaluate the association of folate intake and HNSCC risk, and to characterize the dose–response relationship between HNSCC and folate intake.

The present meta-analysis of 9 studies including 4090 patients and 10,802 controls provided a quantitative estimate of associations between HNSCC risks and folate intake. Our results showed that increased folate intake was associated with risk of HNSCC. The inverse relationships could be detected in subgroup analysis stratified based on countries, anatomical sites, source of controls, folate intake measurement, and NOS quality. Moreover, a dose–analysis meta-analysis indicated that with a 4.3% decrease in HNSCC risk was recorded for increased 100 μg/d folate intake with a linearity model.

Folate is a water-soluble B vitamin and is found in many foods including fruits, vegetables legumes, cereals, and liver. Humans cannot produce folate de novo and need to uptake folate from dietary intake.^[6,36]^ There are 2 main mechanisms that folate deficiency induces carcinogenesis: (1) by inducing complete transformation of dUMP to dTMP, which makes mis-incorporation of uracil into DNA and leads to chromosomal breaks and mutations; and/or (2) by causing abnormal methylation level of DNA, leading to alternations in expression of critical proto-oncogenes and tumor suppressor genes.^[33,34]^ Experiments in vivo referring mice and dogs suggested that increased folate intake alternated DNA methylation and eventually reduced the risks of cancers.^[37,38]^ In addition, the polymorphisms of the 5,10-methylenetetrahydrofolate reductase, which is a critical junction in folate-metabolizing pathway by guiding folate metabolites to DNA methylation pathway and away from the DNA synthesis pathway, may modulate the susceptibility of several cancers.^[14,39]^

Our results in this meta-analysis firstly indicated that folate intake is associated with decreased risk of HNSCC, which is consistent with the role of folate plays in majority of cancers. In addition, a comprehensive dose–response meta-analysis was conducted to quantitate the relationship between dietary folate intake and the risk of HNSCC. Interestingly, compared with low levels of folate intake, high levels of folate intake also make a positive effect on overall survival of HNSCC, which indicated that folate intake is associated with prognosis of HNSCC.^[40]^

In subgroup analysis based on countries, anatomical sites, source of controls, folate intake measurement, and study quality, we observed an overall inverse association between folate intake and HNSCC risk. However, in OPSCC subgroup analysis, only 1 study matched the histological diagnosed squamous cell carcinoma and was included. In the previous study, Galeone et al^[12]^ found that high level of folate intake was associated with decreased risk of oral and pharyngeal cancer (OPC). OPSCC is the major pathological type of OPC, which may support our results. In addition, compared with detecting folate level by Food Frequency Questionnaire, the OR of folate serum level was relative high in our meta-analysis. The plausible reason was that the subgroup analysis of folate serum level only included 2 studies, and the result of 1 study showed no link existed between folate intake and HNSCC risk.^[19]^

Some limitations were significant in our meta-analysis: (1) since the included studies in our meta-analysis were all case-control studies, lack of prospective study, which could affect recall bias and selection bias, restricted the precision of our results; (2) in some subgroup analysis, the numbers of included studies were too small and may make influence on the last conclusions; (3) the analyses in our meta-analysis were pooled data (individual data were not provided), which prevented the further detailed analysis and precise results from being obtained. Hence, our results should be interpreted with caution.

## 5 Conclusions

In conclusion, the results of our meta-analysis indicate that a significant association exists between increased folate intake and decreased HNSCC risk. Dose–response analysis of dietary folate intake indicates that every 100 μg/d folate intake accounts for a 4.3% decrease of HNSCC risk. Furthermore, the present meta-analysis suggests that well-designed and observed prospective studies are necessary for detecting the precise relationship between folate intake and HNSCC risk in the future.
